# Is the experience of chronic pain different in frail older adults? A cross-sectional exploratory study

**DOI:** 10.1055/s-0045-1812302

**Published:** 2025-11-28

**Authors:** Nayara Tasse de Oliveira Cirino, Marcos Paulo Miranda de Aquino, Camila Astolphi Lima, Fânia Cristina dos Santos, Mauricio de Miranda Ventura, Monica Rodrigues Perracini

**Affiliations:** 1 Universidade Cidade de São Paulo, Programa de Mestrado e Doutorado em Fisioterapia, São Paulo SP, Brazil.; 2 Universidade Federal de São Paulo, Escola Paulista de Medicina, Departamento de Medicina, Disciplina de Geriatria e Gerontologia, São Paulo SP, Brazil.; 3 Hospital do Servidor Público Estadual, Ambulatório de Geriatria, São Paulo SP, Brazil.

**Keywords:** Chronic Pain, Frailty, Aged, Pain Measurement

## Abstract

**Background:**

Chronic pain is highly prevalent in frail older adults, resulting in reduced mobility and poor quality of life. However, research on the experience of chronic pain among frail older adults is scarce.

**Objective:**

To compare the experience of chronic pain among frail, prefrail, and non-frail older adults, and to identify associations involving pain measures and frailty syndrome.

**Methods:**

We conducted a cross-sectional study with older adults aged ≥ 60 years presenting chronic pain. The participants were recruited by convenience in specialized outpatient services at public hospitals. Frailty syndrome was identified through the frailty phenotype. The experience of pain was compared among the groups, and we conducted a multivariate logistic regression analysis adjusted for covariates.

**Results:**

Out of the 135 participants, 36.3% were non-frail, 38.5%, prefrail, and 25.2%, frail. Frail older adults presented severe pain more frequently ( *p* = 0.009) and had worse scores for neuropathic pain (mean: 4.1; 95%CI: 3.2–5.1) and depression associated with chronic pain (mean: 9.7; 95%CI: 7.9–11.5) compared with non-frail older adults ( *p* < 0.001). Moreover, frail older adults presented worse multidimensional pain scores (mean: 59.4; 95%CI: 51.7–67.2) compared with non-frail ( *p* = 0.001) and prefrail older adults ( *p* = 0.017). Frail older adults were 3.5-fold as likely to present neuropathic pain, and they presented a 7-fold higher risk of severe pain than non-frail and prefrail older adults.

**Conclusion:**

Frail older adults present severe chronic pain and experience neuropathic pain more frequently. Comprehensive chronic pain assessment and management in this population is critical to achieve active and healthy aging.

## INTRODUCTION

Chronic pain affects 40 to 53% 1 of community-dwelling older adults and is associated with social isolation, disability, reduced quality of life and mobility, increased risk of falls, and depression and anxiety. 2 Older adults with chronic pain commonly need long-term care, creating a significant burden on health and social systems. 3 Chronic pain management is multidimensional and depends on the assessment of underlying diseases and health conditions. 4

Frail older adults present a high prevalence of chronic pain (range: 45–87.5%). 5 Moreover, studies 6 have shown that greater pain intensity might increase the level of frailty. In a study, 7 the chances of developing frailty syndrome among older adults with chronic pain were 3.8- and 24.2-fold higher in those with moderate and severe pain intensity, respectively, compared with older adults without pain. Physiopathological mechanisms related to neuroendocrine deregulation may influence the experience of pain in frail older adults and hinder pain management. 8

**Figure 1:**
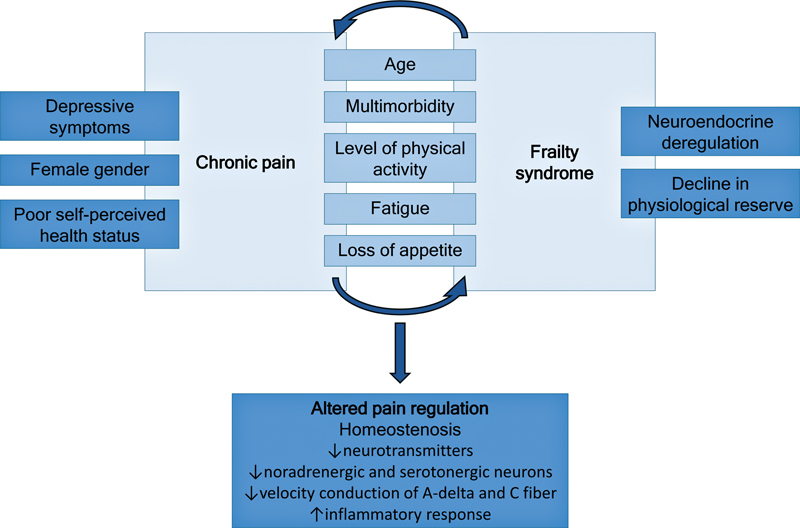
Interaction between chronic pain and frailty syndrome in older adults, which contributes to poor pain regulation.

Chronic pain and frailty syndrome are independent conditions that share several associated factors, such as increasing age, multimorbidity, decreased physical activity level, fatigue, and appetite loss. 9 10 11 The interaction between chronic pain and frailty may exacerbate alterations in pain regulation, which are characterized by homeostenosis, reduced levels of neurotransmitters, reduced activity of noradrenergic and serotonergic neurons, reduced transmission of A-delta and C fibers, and increased inflammatory response. 12 13 14 15 ( Figure 1 ).

Moreover, chronic pain might present neuropathic aspects. Neuropathic pain and its emotional consequences, such as pain-induced depression, represent critical challenges in the clinical management of older adults. Although often neglected and underdiagnosed, these conditions significantly contribute to disability, reduced quality of life, and increased healthcare use. 16 Within the context of frailty, neuropathic pain may reflect central sensitization and a broader neurological vulnerability, reinforcing the importance of integrating pain-related neurological markers into geriatric assessments. 17 Exploring these components may offer valuable insights for neurologists and other specialists involved in the care of older adults with complex pain syndromes.

Despite the high prevalence of chronic pain in older adults with frailty syndrome, studies evaluating chronic pain characteristics in this population are still scarce. The Integrated Care of Older People (ICOPE) framework 18 suggests rating pain severity in older adults at risk or with significant loss of intrinsic capacities but it does not make further recommendations regarding frailty status. The identification of painful experiences in older adults with frailty syndrome enables comprehensive and integrated pain management. In this context, the objective of the current study was to compare the experience of chronic pain (considering intensity, multidimensional aspects, neuropathic components, and pain-induced depression) among frail, prefrail, and non-frail older adults, and to investigate the association involving pain measures and frailty syndrome.

## METHODS

### Study design and participants

The present is an exploratory cross-sectional study. The participants were recruited by convenience in specialized outpatient services at public and university hospitals.

Community-dwelling male and female older adults aged ≥ 60 years and presenting chronic pain (for ≥ 3 months) were included. We excluded participants who were unable to walk, or those who presented communication problems (such as severe hearing loss, aphasia, or difficulty understanding the Portuguese language), postherpetic and/or neoplastic pain, and severe cognitive or visual impairments. The study was approved by the Ethics in Research Committee of Universidade Cidade de São Paulo (under CAAE: 04108318.1.0000.0064), and all participants received information and signed the informed consent form.

### Measures and instruments

#### *Chronic pain*

Pain intensity was assessed using two instruments. The Visual Analog Scale (VAS) 19 was used to investigate pain intensity at the moment of the assessment. The VAS consists of a 10-cm ruler anchored at the extremities by two verbal descriptors, “no pain” and “worst pain possible,” with values classified as no pain (0–4.99 cm), mild pain (5–4.499 cm), moderate pain (4.5–7.499 cm), and severe pain (7.5–10 cm). The participants were instructed to point to the location on the line that best represented pain intensity. The VAS has a good test-retest reliability, which is higher among literate (r = 0.94; *p* < 0.001) than illiterate individuals (r = 0.71; *p* < 0.001). 20 It has excellent reproducibility (intraclass correlation coefficient [ICC] = 0.855) in adults aged > 18 years. 21

The Numeric Rating Scale (NRS), 22 which is composed of 11 (0–10) points, was used to assess pain intensity in the last 7 days. The participants were asked to point to the number that best represented pain intensity, from 0 (no pain) to 10 (worst possible pain). The NRS presents a high test-retest reliability among illiterate (ICC = 0.950) and literate individuals (ICC = 0.960). 20

The pain was evaluated using the Geriatric Pain Measure (GPM), 23 a multidimensional questionnaire that assesses pain in older adults in the sensory-discriminative, affective-motivational, and cognitive-evaluative dimensions. It consists of 22 dichotomous questions (“yes” or “no” answers) that assess pain with ambulation, disengagement due to pain, pain with strenuous activities, and pain with other activities. Two items related to current pain and pain in the previous week are scored categorically on a scale from 0 to 10. The total score is calculated by adding the affirmative responses (1 point each) and the pain intensity (questions 19 and 20). The final score ranges from 0 to 42 and can be adjusted to a scale from 0 to 100 by multiplying the total score by 2.38. Pain severity can be classified as mild (0–30), moderate (31–69), and severe (≥ 70). The internal consistency of the GPM presents a Cronbach α of 0.945 and a good/satisfactory test-retest reliability (ICC = 0.457). 23

Pain-induced depression was evaluated using the Geriatric Psychosocial Assessment of Pain-Induced Depression (GEAP), 24 which is composed of 25 dichotomous questions divided into 3 domains: beliefs about pain, perceived physical limitation, and pain interference in cognition. One point is added for each affirmative answer, and the final score ranges from 0 to 25. The degree of association between pain and depression is classified as absent (0), mild (1–5), moderate (6–9), and severe (≥ 10). The GEAP has excellent inter- (ICC = 0.98) and intraexaminer (ICC = 0.92) reliability. It presents a positive and moderate correlation with the number of depressive symptoms evaluated in the Geriatric Depression Scale (r = 0.590) and the GPM total score (r = 0.495). 25

Neuropathic pain was assessed using the Neuropathic Pain in 4 Questions Questionnaire (Douleur Neuropathique en 4 Questions, DN4, in French), 26 which is composed of 10 items grouped into 4 questions related to pain characteristics, symptoms, sensitive examination, and presence of allodynia. One point is added for each affirmative answer, and the final score ranges from 0 to 10. Scores ≥ 4 indicate neuropathic pain. Test-retest reliability values range from 0.62 to 0.80. 27

### Frailty syndrome

Frailty syndrome was evaluated through the frailty phenotype, 28 consisting of five criteria:

- unintentional weight loss: > 4.5 kg or ≥ 5% of body weight in the previous year;

- self-reported exhaustion, identified by two statements of the Center for Epidemiological Studies Depression Scale; 29

- reduced grip strength: measured using a hand-held dynamometer (SAEHAN, model SH 5001). The cutoff points used for male and female patients were of < 26 kg/f and < 16 kg/f respectively; 30

- slow walking speed: assessed in a 4.60-m corridor, with 2 m for acceleration and deceleration. The average time of 3 measurements was computed and converted into meters/second. The cutoff point of < 0.8 m/s was adopted;

- low physical activity level: measured using three questions: “Did you perform any planned physical activity more than twice a week for more than 30 minutes?,” “Did you do any unplanned walking activity more than twice a week for more than 15 minutes?,” and “Did you do any moderate or vigorous household chores (e.g., washing or mopping the floor, vacuuming, or washing windows or a car) at least once a week for 30 minutes?”. For the criterion to be considered positive, the participant should not have performed any activities with their respective frequency and duration. The participants were classified as frail (presence of three or more criteria), prefrail (one or two criteria), and non-frail (none of the criteria).

### Sample characterization

The following sociodemographic data were evaluated: age, gender, level of schooling, marital status, and living alone. The Functional Comorbidity Index (FCI) 31 assessed the impact of comorbidities on physical function. The Brazilian version of the Older Americans Resources and Services (OARS) Multidimensional Functional Assessment Questionnaire (BOMFAQ) 32 was used to evaluate the degree of difficulty in performing activities of daily living and instrumental activities of daily living. Physical functioning was evaluated using the Short Physical Performance Battery (SPPB), 33 the Four-Square Step Test (FSST), and the Fall Efficacy Scale–International (FES-I). 34

### Statistical analysis

#### *Sample size*

The sample size was calculated a priori according to the mean GPM scores of 20 older adults divided into 3 groups: non-frail (25.98 ± 18.61), prefrail (47.01 ± 17.90), and frail (55.53 ± 31.66). The effect-size was calculated (Cohen's f = 0.360) and, considering a significance level of α = 0.05 and a statistical power of 1-β = 0.90, the optimal number was estimated as 102 participants (34 in each group). The sample size was also calculated regarding the total scores on the VAS, DN4, and GEAP, and values of 24 (Cohen's f = 0.434), 15 (Cohen's f = 0.561), and 16 participants (Cohen's f = 0.543) per group, respectively, were obtained.

The data are expressed as mean ± standard deviation values, unless otherwise stated. Data normality was assessed using the Kolmogorov-Smirnov test. Pain among the groups was compared using the Kruskal-Wallis test or ordinary one-way analysis of variance (ANOVA), and, in case of statistical significance, the Dunn's or the Tukey's post-hoc test was applied to identify differences. Associations involving the groups and the pain measures (VAS, NRS, GEAP, and GPM) were verified using the Chi-squared test.

Stepwise backward multivariate logistic regression models were built to assess the determinants of pain variables in frail compared with non-frail and prefrail older adults. The analysis was adjusted for age, gender, level of schooling, number of medications, number of comorbidities, falls in the previous year, osteoarthritis, osteoporosis, diabetes, peripheral vascular disease, and depression. The goodness-of-fit was verified using the Hosmer-Lemeshow test. Data were analyzed using the IBM SPSS Statistics for Windows (IBM Corp.) software, version 23.0, and the level of significance was set at *p* < 0.05 (two-tailed).

## RESULTS

**Figure 2:**
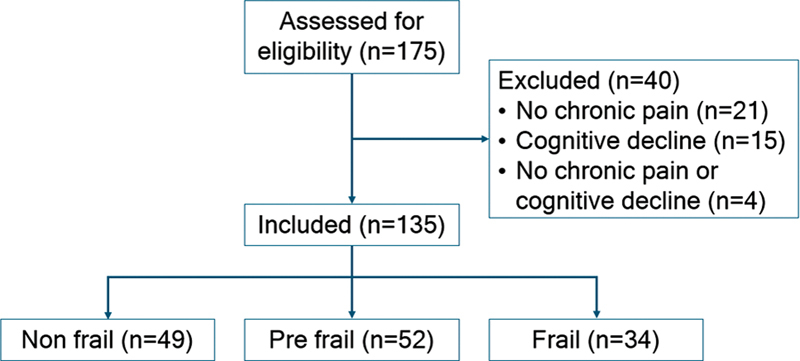
Study flowchart.

We included 135 older adults (85.2% of women) with chronic pain ( Figure 2 ). Their mean age was of 75.2 ± 8.0 (range: 60 to 93) years, and 36.3% (49) of them were classified as non-frail, 38.5% (52), as prefrail, and 25.2% (34), as frail. Table 1 presents the characteristics of the subjects.

**Table 1:** Sample characterization

Variables		Values
Female gender, n (%)		115 (85.2)
Age, n (%)	60–69 years	43 (31.9)
	70–79 years	45 (33.3)
	≥ 80 years	47 (34.8)
Level of schooling, n (%)	Low	99 (73.3)
	Middle	27 (20.0)
	High	9 (6.7)
Medications, median (IQR)		3 (3)
Number of comorbidities (FCI), median (IQR)		4 (3)
	Osteoarthritis, n (%)	77 (57.0)
	Osteoporosis, n (%)	49 (36.3)
	Diabetes, n (%)	50 (37.0)
	Depression, n (%)	31 (23.0)
	Obesity (BMI ≥ 30 kg/m ^2^ ), n (%)	23 (17.0)
Falls in the previous 12 months, n (%)	0	68 (50.4)
	1	24 (17.8)
	≥ 2	43 (31.9)
Perceived risk of falling (FES-I), n (%)	Low	52 (38.5)
	High	83 (61.5)
ADL/IADL limitation (BOMFAQ), n (%)	0	14 (10.4)
	1–3	51 (37.8)
	4–6	34 (25.2)
	≥7	36 (26.7)
Lower limb functionality (SPPB), n (%)	≤ 7	33 (24.4)
	8–12	102 (75.6)
FSST (s), median (IQR)		13.6 (6.4)
Frailty syndrome, n (%)	Non-frail	49 (36.3)
	Prefrail	52 (38.5)
	Frail	34 (25.2)

Abbreviations: ADL, activities of daily living; BMI, Body Mass Index; BOMFAQ, Brazilian version of the Older Americans Resources and Services (OARS) Multidimensional Functional Assessment Questionnaire; IASDL, instrumental activities of daily living; FCI, Functional Comorbidity Index; FES-I, Falls Efficacy Scale–International; FSST, Four-Square Step Test; ; IQR, interquartile range; SPPB, Short Physical Performance Battery.

**Figure 3:**
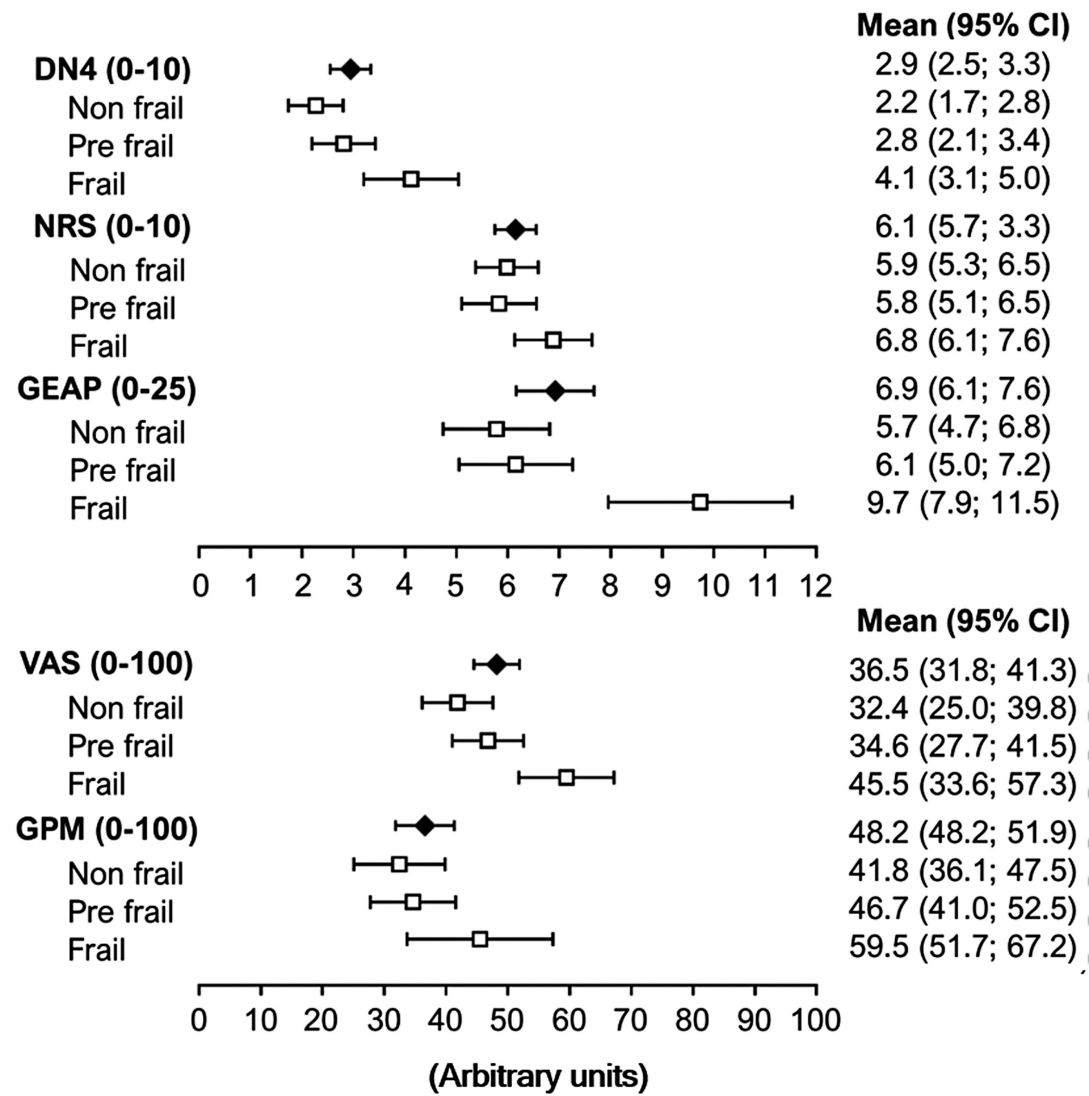
Mean and 95%CI values regarding the Neuropathic Pain in 4 Questions Questionnaire (Douleur Neuropathique en 4 Questions, DN4, in French), the Numeric Rating Scale (NRS), the Geriatric Psychosocial Assessment of Pain-Induced Depression (GEAP), the Visual Analog Scale (VAS), and the Geriatric Pain Measure (GPM) according to the frailty phenotype.

The details on the experience of chronic pain among the groups are shown in Table 2 . Figure 3 shows pain measurements (means and 95%CIs) according to the frailty phenotype. Frail older adults presented significantly more pain sites than the non-frail group, and more pain in the leg ( *p* = 0.001), hip ( *p* = 0.030), elbow, wrist, and hand regions ( *p* = 0.032) than non-frail and prefrail older adults. The prevalence of severe pain at the moment of the assessment was significantly higher ( *p* = 0.009) in frail compared to non-frail and prefrail older adults.

**Table 2:** Pain characteristics among non-frail (NF), prefrail (PF), and frail (F) older adults regarding specialized care ( *n* = 135)

Pain characteristics		Non-Frail ( *n* = 49)	Prefrail ( *n* = 52)	Frail ( *n* = 34)	*p* -value
Number of pain sites*, median (IQR)		2 (2)	2.5 (1)	3 (4)	0.018 (NF versus F); 0.027 (NF versus PF); and 0.232 (PF versus F)
Pain intensity at the moment of the assessment (VAS), median (IQR)		35 (39.5)	35.8 (39.7)	53 (76)	0.132
Pain intensity in the previous week (NRS), median (IQR)		6 (3)	6 (4)	7.5 (4)	0.105
GPM score ^†^ , mean (95%CI)		41.8 (36.1–47.5)	46.7 (41.0–52.5)	59.4 (51.7–67.2)	0.001 (NF versus F); 0.461 (NF versus PF); and 0.017 (PF versus F)
DN4 score*, mean (95%CI)		2.2 (1.7–2.8)	2.8 (2.1–3.4)	4.1 (3.2–5.1)	0.001 (NF versus F); 0.436 (NF versus PF); and 0.022 (PF versus F)
GEAP score*, mean (95%CI)		5.7 (2.7–4.1)	6,1 (5.1–7.2)	9.7 (7.9–11.5)	< 0.001 (NF versus F); 0.891 (NF versus PF); and < 0.001 (PF versus F)
Pain sites, n (%)	Arm	3 (6.1)	4 (7.7)	3 (8.8)	0.894
	Shoulder	22 (44.9)	28 (53.8)	12 (35.3)	0.237
	Elbow, fist, and hand ^‡^	3 (6.1)	11 (21.2)	9 (26.5)	0.032
	Leg ^‡^	7 (14.3)	15 (28.8)	19 (55.9)	0.001
	Hip ^‡^	2 (4.1)	9 (17.3)	8 (23.5)	0.030
	Knee	22 (44.9)	22 (42.3)	9 (26.5)	0.203
	Ankle and foot	8 (16.3)	11 (21.2)	7 (20.6)	0.807
	Cervical	13 (26.5)	7 (13.5)	10 (29.4)	0.146
	Thoracic	7 (14.3)	4 (7.7)	9 (26.5)	0.056
	Low back	24 (50)	29 (55.8)	23 (67.6)	0.240
VAS (0–100 mm) ^‡^ , n (%)	Mild (5–44.99 mm)	35 (71.4)	34 (65.4)	15 (44.1)	0.009
	moderate (45.00–74.99 mm)	11 (22.4)	14 (26.9)	9 (26.5)	
	severe (≥ 75.00 mm)	3 (6.1)	4 (7.7)	10 (29.4)	
GPM (0–100) ^‡^ , n (%)	mild (0–30.9)	17 (34.7)	12 (23.1)	3 (8.8)	0.001
	moderate (31–69.9)	30 (61.2)	33 (63.5)	12 (35.3)	
	severe (≥ 70)	2 (4.1)	7 (13.5)	12 (35.3)	
Presence of neuropathic pain (DN4), n (%) ^‡^		11 (22.4)	19 (36.5)	19 (55.9)	0.004
GEAP (0–25) ^‡^ , n (%)	absent (0)	1 (2)	2 (3.8)	0 (0)	0.010
	mild (1–5)	24 (49.0)	24 (46.2)	10 (29.4)	
	moderate (6–9)	16 (32.7)	18 (34.6)	9 (26.5)	
	severe (≥ 10)	8 (16.3)	8 (15.4)	15 (44.1)	

Abbreviations: DN4, Douleur Neuropathique en 4 Questions (Neuropathic Pain in 4 Questions) questionnaire; GEAP, Geriatric Psychosocial Assessment of Pain-Induced Depression; GPM, Geriatric Pain Measure; IQR, interquartile range; NRS, Numerical Rating Scale; VAS, Visual Analogue Scale.

Notes: *Kruskal-Wallis test. ^†^ Ordinay one-way analysis of variance (ANOVA). ^‡^ Chi-squared test.

The multidimensional impact of pain was significantly worse in frail than in non-frail and prefrail older adults ( *p* = 0.001). Compared with the non-frail and prefrail groups, frail older adults presented greater tiredness or fatigue ( *p* = 0.021), they stopped working or performing other activities due to pain ( *p* = 0.008), they stopped doing something they liked and reduced the amount of work and other activities ( *p* = 0.002), they reported requiring greater effort while performing their activities ( *p* = 0.004), they needed the help of others due to pain ( *p* < 0.001), they felt sad or depressed due to pain, and they reported more pain when walking long distances ( *p* < 0.001), less than 1 block ( *p* < 0.001), or while performing activities of moderate intensity ( *p* = 0.039). Frail older adults also presented more pain when bathing and dressing ( *p* = 0.015) and reported that pain prevented them form participating in religious activities ( *p* = 0.022) or any other activity ( *p* = 0.010) ( **Supplementary Material Table S1** , available at: https://www.arquivosdeneuropsiquiatria.org/wp-content/uploads/2025/07/ANP-2024.0170-Supplementary-Material.docx ).

The DN4 scores were significantly higher in the frail group compared to the non-frail and prefrail groups, mainly due to more sensation of tingling ( *p* = 0.036), pins and needles ( *p* = 0.017), numbness ( *p* = 0.039), hypoesthesia to touch ( *p* < 0.001), and hypoesthesia to pinprick ( *p* = 0.001) ( **Supplementary Material Table S2** ).

Chronic pain induced-depression was higher in frail than in non-frail and prefrail older adults ( *p* <0.001). Pain affected the appetite ( *p* = 0.002) of frail older adults, the performance of pleasant activities ( *p* = 0.012), and made them feel like they could not go on living ( *p* = 0.002) and that they were worthless ( *p* = 0.008). It also prevented this group from ever being happy again ( *p* = 0.023), from returning to activities they used to perform ( *p* = 0.019), from controlling their feelings ( *p* = 0.006), and led them to stop doing everything ( *p* = 0.037) compared with non-frail and prefrail older adults ( **Supplementary Material Table S3** ).

The covariate-adjusted multivariate regression analysis presented in **Table 6** ( **Supplementary Material 2** available at https://www.arquivosdeneuropsiquiatria.org/wp-content/uploads/2025/10/ANP-2024.0170-Supplementary-Material-2.docx ) showed that frail older adults are 3.53 as likely to present neuropathic pain (odds ratio [OR] = 3.53; 95%CI = 1.37–9.09; *p* = 0.009) and 7.46 as likely to present severe pain (OR = 7.46; 95%CI = 2.20–25.29; *p* = 0.001) compared with non-frail and prefrail older adults.

## DISCUSSION

Our findings extend previous evidence that frailty is strongly associated with greater pain burden. Compared with the non-frail and prefrail groups, frail older adults presented more pain sites and a 5-fold higher prevalence of severe pain. These results are in line with those of Shega et al. (2012) 12 and Rodríguez-Sánchez et al. (2019), 35 who also reported that frailty is significantly associated with increased pain intensity and number of pain sites, especially in musculoskeletal conditions. In the current study, frail older adults were 3.5 and 7.4 times more likely to present neuropathic pain and severe pain intensity, respectively, compared with non-frail and prefrail older adults.

Regarding pain sites, frail older adults presented a higher frequency of pain in the elbow, wrist, hand, and lower limbs. Studies indicate that chronic pain is associated with other diseases such as osteoarthritis (OA), 36 and lower limb pain due to OA is associated with frailty, which may explain its high incidence (that is, 57% of the sample was diagnosed with OA, and 68.8% were prefrail and frail). Conversely, low back pain was not different among the groups, despite its high incidence (56.3%). Curiously, pain in the elbow, wrist, and hand was more reported by frail older adults, probably due to the presence of OA in these joints. Previous studies 27 indicate that approximately half of frail older adults present pain at multiple sites, which could be explained by multiple morbidities, the coexistence of other geriatric syndromes, functional limitation, and physical inactivity. The presence of pain at multiple sites is associated with worse health outcomes, such as the risk of fracture, depression, and worse physical health, quality of life, and sleep. 25 35 The present study warns that these outcomes may worsen in frail older adults due to multiple morbidities and chronic pain.

Several prospective studies have indicated that the higher the frequency, intensity, and number of pain sites, the higher the risk of developing or aggravating the frailty syndrome. 37 A cross-sectional study 12 using data from the Canadian Study of Health and Aging (CSHA) found that frail older adults were 5.5 times more likely to present moderate to severe pain than non-frail participants. The relationship between severe chronic pain and frailty can be explained by the pain homeostenosis effect, which is the decline in intrinsic abilities and age-related functional skills that reduces the ability to cope with stressors, hindering pain regulation and leading to frailty development. 12 However, the complex interrelationship between frailty syndrome and pain is still little understood. A recent study showed an association between moderate pain and frailty level only in the 65-year-old group (OR = 3.00; 95%CI = 1.30–6.60), indicating an effect of age. Interestingly, neither the presence of comorbidities nor the decline in cognitive function exerted a mediating effect on pain and frailty. Prospective studies are necessary to understand the underlying mechanisms explaining this effect.

Nevertheless, pain intensity in frail older adults is not a consensus, since no differences were observed among individuals with frailty using continuous scales. 38 39

Our findings highlight the significant multidimensional impact of pain in frail older adults, affecting basic self-care activities such as bathing and dressing, and reducing engagement in meaningful social activities, including religious participation. These results are consistent with those of Brown et al. (2019), 40 who also observed that pain in frail individuals severely compromises daily functioning and quality of life. The use of the GPM in both studies reinforces that pain among frail older adults extends beyond physical discomfort, encompassing emotional and functional domains. This emphasizes the need for multidimensional pain management approaches specifically tailored to this population.

Frail older adults also presented a higher likelihood of having neuropathic pain, which has been associated with musculoskeletal pain and factors linked to frailty syndrome, such as the female gender, advanced age, pain site, low income, and the presence of other chronic comorbidities. 40 Neuropathic pain from peripheral or central somatosensory system lesions or dysfunction is characterized by limiting symptoms. However, only a small percentage of older adults receive adequate pharmacological treatment for neuropathic pain. 41 Moreover, in the present study, higher chronic pain-induced depression scores were observed in frail older adults compared with the non-frail and prefrail groups. Depression is associated with the female gender, severe pain intensity, worse cognition, coexisting diseases, functional impairment, and low socioeconomic level. Epidemiological studies 24 have shown a bidirectional relationship between chronic pain and depression, which contributes to identifying common pathogenic factors between them, represented by chronic neuroinflammation. Our results suggest that this relationship exists, even though a causal relationship was not established.

The co-occurrence of neuropathic pain and pain-induced depression among frail older adults suggests the presence of a high-risk clinical subgroup with complex neuropsychiatric vulnerability. While no specific subgroup analysis was performed in the current study, identifying these patients—based on validated tools such as the DN4 and GEAP—has practical implications for neurologists. Future studies may benefit from examining this subgroup more closely, as these combined symptoms may indicate a more severe or treatment-resistant pain profile requiring specialized care.

In the sample of the present study, the prevalence of diabetes increased with the frailty level, corroborating epidemiological trends showing that diabetes is highly prevalent among older adults in Brazil. 42 This association can be attributed to a range of interrelated mechanisms. Diabetes is frequently accompanied by sarcopenia, which directly contributes to the physical dimension of frailty. 43 In addition, chronic low-grade inflammation, which is commonly observed in individuals with diabetes, can exacerbate muscle degradation and impair physical function. Diabetic neuropathy, another common complication, compromises sensory input and balance, thereby increasing the risk of falls and promoting functional decline. 44 45

Moreover, metabolic dysregulation resulting from insulin resistance and hyperglycemia hinders energy production and muscle efficiency, further aggravating weakness and fatigue. 46 Cognitive impairments associated with diabetes may also interfere with self-care behaviors, including medication adherence and physical activity, which are essential to maintain independence in older age. 47 Finally, polypharmacy is prevalent among older adults with diabetes, increasing the likelihood of adverse drug interactions and side effects that may intensify frailty. 48 Although diabetes was included as an adjustment variable in our multivariate models, its potential contribution to chronic pain, neuropathy, and overall functional decline in frail individuals deserves further exploration to better understand these complex and multifactorial interactions.

Socioeconomic disparities, particularly low levels of schooling, were prevalent among the participants of the current study, and they may have influenced both the experience and the reporting of pain. Limited health literacy can affect older adults' ability to communicate symptoms accurately, adhere to treatments, and engage in self-care, intensifying frailty and pain. Moreover, unequal access to healthcare services and resources across regions in Brazil may contribute to late diagnoses, undertreatment of chronic pain, and progression toward greater physical vulnerability. These factors should also be considered when interpreting the results and planning targeted interventions for older adults in resource-limited settings. 49

## Limitations

The current study has limitations. First, the sample was mostly composed of women, limiting the representativeness of our findings to the general population. Second, frailty was determined using the phenotype proposed by Fried et al. 28 (2001), which is markedly composed of physical criteria. Third, data on the duration of pain and the length of treatment among participants was not collected. These factors could influence pain perception, functional impairment, and the observed associations with frailty. Future investigations should consider the temporal dimension of chronic pain, as prolonged exposure to untreated or undertreated pain may lead to central sensitization, emotional distress, and resistance to conventional interventions. 50 Moreover, the lack of detailed information on pharmacological treatment and access to specialized care might have limited the scope of the present study. Medication regimens, including analgesics and adjuvant therapies such as antidepressants or anticonvulsants, may directly influence pain severity, mood, and physical function. 51 Additionally, disparities in access to neurologists, geriatricians, pain clinics, or rehabilitation services are common in the Brazilian public health system, and they may shape the experience and the outcomes of chronic pain in older adults. 52 Future studies should address these variables to support more equitable and individualized clinical decision-making.

## Clinical implications

Frail older adults experience more intense and complex chronic pain, including neuropathic components and pain-induced depression. These results suggest a complex neuropsychological profile of pain in frailty, which may comprise central sensitization and emotional distress. Such profiles are clinically-relevant for neurologists, who often manage older patients with overlapping neurological and pain-related disorders. Early identification of neuropathic features may facilitate more targeted interventions to prevent further functional decline. Our findings highlight the need for comprehensive pain assessment and targeted interventions in frail older adults to mitigate frailty and promote healthy aging.
